# NAD World 3.0: the importance of the NMN transporter and eNAMPT in mammalian aging and longevity control

**DOI:** 10.1038/s41514-025-00192-6

**Published:** 2025-01-27

**Authors:** Shin-ichiro Imai

**Affiliations:** 1 Department of Developmental Biology, Department of Medicine (Joint), Washington University School of Medicine, St. Louis, Missouri USA; 2 Institute for Research on Productive Aging (IRPA), Tokyo, Japan

**Keywords:** Ageing, Translational research

## Abstract

Over the past five years, systemic NAD^+^ (nicotinamide adenine dinucleotide) decline has been accepted to be a key driving force of aging in the field of aging research. The original version of the NAD World concept was proposed in 2009, providing an integrated view of the NAD^+^-centric, systemic regulatory network for mammalian aging and longevity control. The reformulated version of the concept, the NAD World 2.0, was then proposed in 2016, emphasizing the importance of the inter-tissue communications between the hypothalamus and peripheral tissues including adipose tissue and skeletal muscle. There has been significant progress in our understanding of the importance of nicotinamide mononucleotide (NMN), a key NAD^+^ intermediate, and nicotinamide phosphoribosyltransferase (NAMPT), particularly extracellular NAMPT (eNAMPT). With these exciting developments, the further reformulated version of the concept, the NAD World 3.0, is now proposed, featuring multi-layered feedback loops mediated by NMN and eNAMPT for mammalian aging and longevity control.

## Introduction

The parable of the blind men and an elephant is well-known and often used as a metaphor to caution how limited our knowledge and experience would be. At the end of this famous story, there was a king who told blind men that what they were touching was actually a big animal. Basically, what blind men were describing about the elephant was all correct. However, the real challenge in this famous parable is: if there is no king to tell the truth, how could they know the whole picture of the elephant simply based on their own observations? Arguing who is right and who is wrong would not be so helpful and constructive because there is no definitive truth in their minds. Probably, the best possible approach would be to assume that everybody is correct and try to build an image that could possibly accommodate all observations. In this approach, the key is to find a common thread which could stitch most of, if not all, pieces of knowledge up together.

Over the past decade, our understanding of the mechanisms of aging has significantly advanced. We now know several important signaling pathways and regulators, including insulin/insulin-like growth factor 1 (IGF-1) signaling, mechanistic target of rapamycin (mTOR) signaling, and the sirtuin family of NAD^+^-dependent protein deacetylases/deacylases. New cellular and molecular processes, such as cellular senescence, autophagy, and proteostasis, have been found to play important roles in the regulation of aging and longevity in different model organisms. Nonetheless, just like the metaphor mentioned above, we still do not have a whole picture of what exactly aging is and how exactly longevity is determined. Unfortunately, we do not have a mighty king to tell us the truth. Then, would it be worth trying to find a common thread that could allow us to build a meaningful image of this big, complicated elephant?

The very first attempt to have such a meaningful image for the regulation of aging and longevity resulted in the introduction of a concept named the “NAD World” in 2009^1–3^. The NAD World is a systemic regulatory network that connects NAD^+^ metabolism, biological rhythm, and aging and longevity control in mammals. In the original NAD World concept, two critical components were proposed to drive the NAD World: The mammalian NAD^+^-dependent protein deacetylase SIRT1 and the key NAD^+^-biosynthetic enzyme NAMPT (nicotinamide phosphoribosyltransferase)^1,2^. While NAMPT generates a circadian oscillation of NAD^+^ production in multiple tissues^4,5^, SIRT1 responds to NAD^+^ availability and regulates many fundamental cellular and physiological processes, including transcription, DNA repair, stress response, metabolism, circadian rhythm, and aging^3,6^. Through these coordinated functions, SIRT1 and NAMPT control the system dynamics of the NAD World and determine the process of aging and eventually, lifespan. The most important prediction from the concept of the NAD World was that the driving force of aging is the systemic decline in NAD^+^ levels. Fifteen years after the first introduction of this concept, the notion that NAD^+^ decline is a key driving force of aging has been accepted as a consensus in the field of aging research^7–11^. Additionally, the functional connection between NAD^+^ and other aging-regulatory pathways and key cellular processes has now become an intensive research topic^12–14^.

The concept of the NAD World was then reformulated as the NAD World 2.0 in 2016, based on significant progress in the field over seven years^6^. In the NAD World 2.0, three key tissues have been identified: The hypothalamus as the control center of aging, skeletal muscle as a mediator, and adipose tissue as a modulator. The details of the NAD World 2.0 were described previously^6^. Among several predictions from the NAD World 2.0, the most critical one is that the secretion of extracellular NAMPT (eNAMPT) from adipose tissue is a key inter-tissue communication between the hypothalamus and adipose tissue in mammalian aging and longevity control. A related prediction is the importance of nicotinamide mononucleotide (NMN), a key NAD^+^ intermediate and the product of the NAMPT enzymatic reaction, in the maintenance of biological robustness. Intriguingly, both predictions have so far been demonstrated, introducing new critical elements into the NAD World 2.0^15–25^. Therefore, in this review article, I would like to discuss these two critical elements, eNAMPT and NMN, and try to stitch these new pieces of knowledge together, taking NAD^+^ as a common thread, into a further reformulated version of the NAD World concept, namely, the NAD World 3.0.

## Systemic NAD^+^ decline is a common thread that drives aging

In the past decade, a significant body of evidence has demonstrated that systemic decreases in NAD^+^ levels drive the age-associated physiological decline in tissue functions and the pathogenesis of age-associated diseases. A number of excellent review articles have already summarized these critical findings that support the notion that systemic NAD^+^ decline is a key driving force of aging^7–11^. This NAD^+^ decline appears to be caused by two underlying molecular events: decreased NAD^+^ biosynthesis^23,24,26–28^ and increased NAD^+^ consumption^29–33^. It is still technically very challenging to measure NAD^+^ flux accurately in vivo. However, one study has recently compared NAD^+^ flux between young and old mice by infusing four deuterium-labeled isotopic nicotinamide, suggesting the maintenance of absolute NAD^+^ fluxes with age^33^. Whereas this study provides critical insight into systemic changes in NAD^+^ flux, many other details, including cellular and tissue heterogeneity, systemic NAD^+^ biosynthesis, and potential feedback regulations, remain to be elucidated to allow us to evaluate accurate NAD^+^ flux. With these future challenges in our minds, it should be emphasized that those two molecular events appear to be two sides of the same coin, namely, chronic, low-grade inflammation^24,26,31,32^.

Our body is constantly exposed to exogenous pathogens and toxic substances. For example, the entire world has gone through the historic COVID-19 pandemic. This new coronavirus, Severe Acute Respiratory Syndrome Coronavirus 2 (SARS-CoV-2), often triggers an excessive immune response called a cytokine storm, elevating circulating cytokine levels, such as interleukin-1β (IL-1β), interleukin-6 (IL-6), and tumor necrosis factor (TNF), and causing systemic inflammatory symptoms and multi-organ dysfunction and eventually, death if not treated adequately^34^. Although this is an extreme case, there are many other already-existing viruses and pathogens that cause a wide variety of inflammatory reactions in different organs^35,36^. Other exogenous stimuli, such as exposures to ultraviolet (UV), allergens, and environmental toxins, can also cause significant inflammatory reactions^37,38^. Additionally, intrinsic signals triggered by metabolic processes and certain cellular responses cause inflammatory responses. Excessive lipid accumulation in lipid droplets, a lipid-storing organelle that responds to a variety of cellular stresses^39^, promotes the secretion of inflammatory mediators^40,41^. Indeed, lipid droplet-accumulating microglia found in the aging brain exhibit impaired phagocytosis and excessive release of pro-inflammatory cytokines, such as IL-1β, IL-6, TNF-α, CCL3, and CXCL10^42^. Because SIRT3 promotes the breakdown of lipid droplets through the activation of AMPK^43^, a potential connection between NAD^+^, sirtuins, and lipid droplet formation may play a significant role in inflammatory responses. Different mechanisms of these inflammatory reactions in response to each stimulus have been extensively investigated, and the importance of chronic, low-grade inflammation has been realized for the pathogenesis of age-associated tissue dysfunctions and diseases^44–46^.

**Fig. 1:**
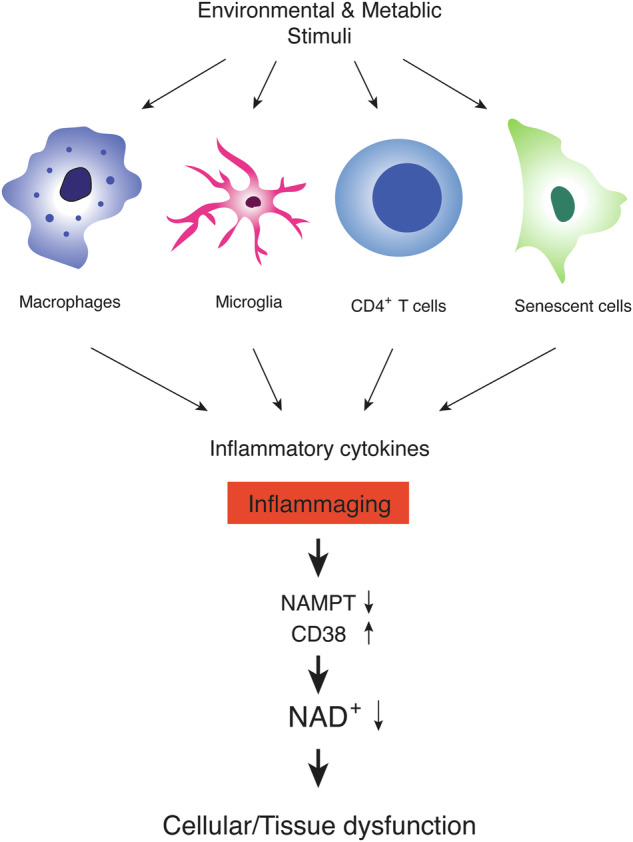
Chronic, low-grade inflammation drives systemic NAD^+^ reduction. Environmental, exogenous stimuli, including pathogens, allergens, ultraviolet (UV), and toxic substances, and metabolic, endogenous stimuli, including lipids, misfolded proteins, and cellular debris, activate specific immune cells, such as macrophages, microglia, and T lymphocytes. These activated immune cells secrete a variety of inflammatory cytokines. On the other hand, senescent cells, which accumulate over age in multiple tissues, also show the senescence-associated secretory phenotype (SASP), secreting many inflammatory cytokines. The constant exposure to these inflammatory cytokines, often called “inflammaging”, decreases the expression of NAMPT, a key NAD^+^ biosynthetic enzyme, and increases the expression of CD38, a major NAD^+^ glycohydrolase, both resulting in a significant reduction in NAD^+^ levels at a systemic level. Such systemic NAD^+^ reduction leads to cellular and tissue dysfunction, causing age-associated pathophysiologies.

Chronic, low-grade inflammation, often called “inflammaging”, is induced by the recurrent activation of specific immune cells, such as macrophages, microglia, and T lymphocytes, and the generation of senescent cells, both of which lead to the secretion of inflammatory cytokines (Fig. 1). For example, macrophages stimulated with lipopolysaccharide show the depletion of NAD^+^ and increased expression of *Nampt*^47^. The depletion of NAD^+^ is caused by reactive oxygen species-induced DNA damage and the resultant activation of poly (ADP-ribose) polymerase (PARP)^47^. On the other hand, the upregulation of *Nampt* expression contributes to the maintenance of NAD^+^ pools, which enhances glyceraldehyde-3-phosphate dehydrogenase activity and, thereby, the Warburg metabolism in inflammatory macrophages^47^. In CD4^+^ T lymphocytes, the deficiency of mitochondrial transcription factor A (*Tfam*) causes mitochondrial dysfunction and increased expression of cytokines, such as IFN-γ and IL-1β, recapitulating features in aged CD4^+^ T cells^48,49^. Indeed, mice with *Tfam*-deficient T cells recapitulate multiple aging-related tissue dysfunctions with elevated circulating cytokine levels, which are partially rescued by increasing NAD^+^ levels^50^, suggesting the involvement of reduced NAD^+^ availability in these mice. Those inflammatory macrophages and CD4^+^ T cells with mitochondrial dysfunction secrete a variety of cytokines and stimulate other cell types. In primary hepatocytes, TNF-α decreases the expression of *Nampt* and their NAD^+^ levels^24^. In adipocytes, inflammatory cytokines increase the expression of CD38, one of the major NAD^+^ glycohydrolases, and decrease their NAD^+^ levels^31,32^. Such NAD^+^ decline could also reduce NAD^+^-dependent sirtuin activities^51–57^, potentially further accelerating the process of chronic inflammation through the activation of NF-κB function and NLRP3 inflammasome by reducing SIRT1/6 activities^58,59^ and SIRT2 activity^60^, respectively.

Senescent cells, which accumulate in multiple tissues during aging, also contribute to inflammaging. Senescent cells exhibit a characteristic feature called the senescence-associated secretory phenotype (SASP), namely, the secretion of a variety of bioactive molecules including pro-inflammatory cytokines^61^. Interestingly, long interspersed element (LINE-1/L1) retrotransposons become derepressed during cellular senescence, inducing a type-I interferon (IFN-I) response^62,63^. Lamivudine, a nucleoside reverse transcriptase inhibitor, blocked the synthesis of cytoplasmic L1 DNA, reduced the IFN-I response, and inhibited the later SASP response (such as induction of CCL2, IL-6 and MMP3), but not the early SASP response (such as IL-1β)^62^. Indeed, treatment of aged mice with lamivudine suppresses the IFN-I response and inflammaging in several tissues^62^. The suppression of L1 re-expression or misexpression is normally mediated by SIRT6, another well-studied member of the mammalian sirtuin family^63,64^. Given that SIRT6 also requires NAD^+^ for its enzymatic activity, it is conceivable that tissue NAD^+^ decline accelerates the re-expression or misexpression of L1 transcripts, forming a negative spiral towards inflammaging^63^.

All these cells, including macrophages, microglia, T lymphocytes, and senescent cells, secrete pro-inflammatory cytokines and are speculated to trigger the cascade of NAD^+^ decline by either decreasing NAD^+^ biosynthesis or increasing NAD^+^ consumption (Fig. 1). Thus, restoring NAD^+^ levels systemically is expected to suppress the effects of inflammation on tissue functions. Indeed, NMN and nicotinamide riboside (NR), two major NAD^+^ boosters, have been demonstrated to suppress inflammatory reactions in a variety of pathophysiological conditions^24,65–69^. Nonetheless, there has so far been no report suggesting that NAD^+^ would function as a broad immunosuppressant, implicating some feedback mechanism that keeps NAD^+^ levels and downstream regulatory pathways within a physiological, homeostatic range. Taken together, inflammaging caused by a variety of extrinsic and intrinsic factors causes a vicious cycle of NAD^+^ decrease, inducing an age-associated functional decline in multiple tissues. Therefore, it will be of great importance to further investigate how exactly those cell types cause inflammaging over time and elucidate how NAD^+^ decrease triggered by inflammaging induces age-associated functional decline at a systemic level.

## NAD^+^ decline drives energetic shifts in the mitochondria and global epigenetic changes in the nucleus

**Fig. 2:**
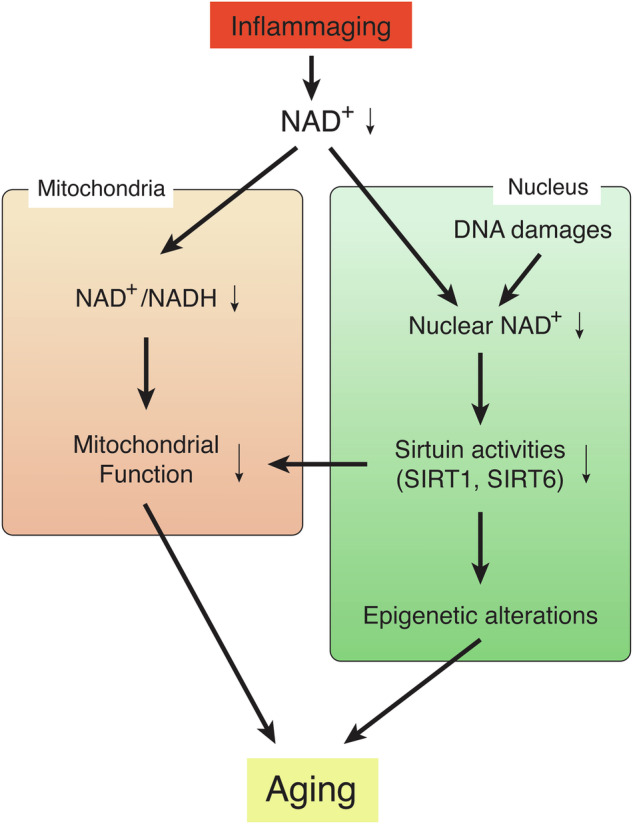
NAD^+^ decline causes mitochondrial dysfunction and global epigenetic changes. Systemic NAD^+^ decline, triggered by the phenomenon called “inflammaging”, induces two critical events: (1) Reduction in the NAD^+^/NADH redox state in the mitochondria, leading to mitochondrial dysfunction, and (2) global epigenetic changes in the nucleus, leading to age-associated transcriptomic changes and functional decline. These two events are not mutually exclusive, and a specific nuclear-mitochondrial communication might contribute to age-associated mitochondrial dysfunction. This signal is triggered by nuclear NAD^+^ decline, which is also caused by DNA damage, and also mediated by sirtuin family members, such as SIRT1 and SIRT6. These two events causally induce a variety of aging phenotypes in a tissue-specific manner.

When tissue NAD^+^ levels decrease over age, the NAD^+^/NADH redox state could be affected (Fig. 2)^51,70,71^. In the human brain, for instance, ultrahigh-field (7 T) in vivo ^31^P magnetic resonance spectroscopy revealed a gradual, significant decline in the NAD^+^/NADH redox state, associated with decreasing NAD^+^ levels and increasing NADH levels during healthy aging^71^. Similar results have been reported for NAD^+^ levels and the NAD^+^/NADH ratio in human plasma samples from the ages ranging from 20 to 87 years^70^. A recent mass spectrometry study also reported a decreased NAD^+^/NADH ratio in the brain, skeletal muscle, and jejunum in aged mice^33^. Such changes suggest a significant shift towards slower oxidative phosphorylation in the mitochondria, resulting in reduced ATP production. A similar reduction in in vivo rates of mitochondrial oxidative and phosphorylation activity was also demonstrated in human-aged skeletal muscle, most likely contributing to the pathogenesis of insulin resistance^72^. While the reduction in the NAD^+^/NADH redox state, driven by age-associated NAD^+^ decreases, leads to mitochondrial dysfunction, it has been suggested that a more specific nuclear-mitochondrial communication might contribute to age-associated mitochondrial dysfunction (Fig. 2). Interestingly, the mitochondrially encoded components of the oxidative phosphorylation (OXPHOS) system are significantly decreased during aging, which is driven by the reduction in nuclear NAD^+^ levels and SIRT1 activity^73^. SIRT1 regulates mitochondrially encoded genes by activating the *TFAM* (mitochondrial transcription factor A) promoter through hypoxia-inducible factor 1 (HIF-1α) and c-Myc. Increasing NAD^+^ levels by NMN can restore the expression of mitochondrially encoded OXPHOS genes through SIRT1-HIF-1α-c-Myc pathway^73^. Such age-associated dysregulation of the mitochondrially encoded OXPHOS genes appears to occur in skeletal muscle and the heart, but not in the liver, white adipose tissue, and the brain^73^. Therefore, the importance of this specific nuclear-mitochondrial communication might be tissue-dependent.

The age-associated reduction in nuclear NAD^+^ levels and sirtuin activities also cause global epigenetic changes in the nucleus (Fig. 2)^51,57,74^. In the aged mouse brain, two-thirds of SIRT1-bound genes that can be depressed by oxidative stress are found to be depressed^74^. Oxidative stress causes a redistribution of SIRT1 through its recruitment to the DNA damage sites and transient increases in its chromatin association. In such DNA-damaging conditions, poly(ADP-ribose)polymerase-1 (PARP-1) is activated so that nuclear NAD^+^ is consumed, leading to decreased SIRT1 activity^29^. Thus, it is conceivable that both events, reduction in nuclear NAD^+^ levels and redistribution of SIRT1, underlie age-associated global gene expression changes, although these events may occur in a tissue/region-specific and/or cell type-specific manner^17^. Another mammalian sirtuin family member, SIRT6, may also be involved in such epigenetic changes. SIRT6 physically interacts with a chromatin remodeler SNF2H, and both are recruited to DNA damage sites^75^. SIRT6 is also important to suppress the expression of L1 retrotransposons through the interaction with KRAB-associated protein 1 (KAP1), heterochromatin protein 1α, and methyl CpG binding protein 2 (MeCP2)^64^. Both oxidative stress and aging induce the expression of L1 in cells and tissues, respectively, which is recapitulated in *Sirt6* KO mice^63,64^. Interestingly, these findings provide strong support to the “Heterochromatin Island” hypothesis, proposed by Kitano and myself a quarter century ago, in which the dynamic reorganization of global transcriptionally repressive structures, termed “heterochromatin islands”, is a driving force of cellular and organismal aging^1,76^. In the “Heterochromatin Island” hypothesis, SIR2 family proteins were predicted to play a key role in reorganizing “heterochromatin islands” and connecting their global changes to aging. This predicted connection between sirtuins, epigenetic regulation, and aging has been demonstrated in different experimental models^22,74,77–79^. Nonetheless, it is still unclear whether the reduction in nuclear NAD^+^ levels, which could be induced by inflammaging, and reorganization of “heterochromain islands”, which could be caused by DNA damages, are functionally connected or independent events to drive age-associated pathophysiologies. Most recently, it has been proposed that erosion of the epigenetic landscape, induced by DNA double-strand breakages, is a cause of mammalian aging^80^. Given that repairing DNA double-strand breakages also consumes nuclear NAD^+^, it is conceivable that the maintenance of nuclear NAD^+^ could delay aging and promote lifespan through the prevention of age-associated epigenetic alternation. In this regard, it is interesting that NAD^+^ boosting by NR was reported to delay senescence of neural and melanocyte stem cells and cause a moderate lifespan extension (5%) when given at an old age^81^, whereas no lifespan-extending effect was observed when NR administration started in young mice^82^. Recently, it has been reported that orally administered NR is degraded to nicotinamide in the gut, increasing circulating nicotinamide levels considerably^83,84^, and nicotinamide does not increase lifespan when started at 12 months of age in mice^85^. Given that high concentrations of nicotinamide inhibit sirtuin activities^54^, the balance between positive and negative effects (NAD^+^ increase vs. nicotinamide increase) could affect the final outcome of each NAD^+^-boosting intervention. NMN, another NAD^+^ intermediate, also produces nicotinamide when orally administered^21^. Although no head-to-head comparisons between NMN and NR pharmacokinetics have ever been reported, NMN transport happens quite rapidly, in a minute order, as discussed in the next section, whereas NR transport appears to be much slower, most likely in an hourly order^83,84,86^. This kinetic difference is critical because orally administered NMN can be transported intact before NMN degrades to nicotinamide, whereas NR is basically subjected to degradation, being converted to nicotinamide and then to nicotinic acid in the gut^83,84^. These characteristics of NMN, a new important addition to the NAD World 3.0, will be discussed in detail in the following sections.

## Slc12a8, an NMN transporter, is a new player in the NAD World

When NAD^+^ levels decline, cells trigger a mechanism by which they try to maintain NAD^+^ levels. This is how exactly we were led to the discovery of a novel NMN transporter, Slc12a8^15^. In our previous studies, we recognized that when reducing NAD^+^ levels with FK866, a potent NAMPT inhibitor, in several primary cells including hepatocytes, pancreatic islets, and neural stem cells, FK866-treated cells tended to respond to NMN administration to a greater extent than FK866-untreated controls, always showing larger NAD^+^ restoration^19,24,87^. Thus, we came up with a hypothesis that cells upregulate the expression of a putative molecule that functions as an NMN transporter. Based on this hypothesis, we searched for a gene that encodes a transmembrane protein and is upregulated by FK866 treatment. Surprisingly, we found one gene that met these criteria, that is, *Slc12a8*. Extensive biochemical and physiological characterizations of Slc12a8 led us to the conclusion that Slc12a8 is an NMN transporter^15^. First, abrogation or deficiency of *Slc12a8* in primary cells or in the small intestine significantly decreases the uptake of NMN, reducing NAD^+^ levels. For example, in *Slc12a8*-deficient primary hepatocytes, the uptake of isotopically doubly labeled NMN detected by mass spectrometry was reduced by ~90% within 5 min, compared to control wild-type hepatocytes. No conversion of isotopically doubly labeled NMN to NR was detected within 5 min, and the uptake of isotopically doubly labeled NR was not affected. Additionally, NAD^+^ levels were significantly decreased in the jejunum of intestinal *Slc12a8*-knockdown mice, compared with control mice, after oral NMN administration. Second, overexpression of full-length *Slc12a8* in NIH3T3 fibroblasts, a cell line that expresses a defective *Slc12a8* transcript (unpublished finding) and thus exhibits negligible NMN transport, enables these cells to transport NMN and increase cellular NAD^+^ levels. Overexpression of *Slc12a8* in the small intestine of young wild-type mice also significantly increases NAD^+^ levels, whereas the small intestine of *Slc12a8*-deficient mice shows a reduction in NMN uptake and NAD^+^ levels. Third, the biochemical characterization using Slc12a8-containing proteoliposomes revealed that Slc12a8 is specific to NMN, not to NR and other related compounds, and that its *K*_*m*_ (34 μM) is consistent with a reported range of NMN concentrations in mice^15,88–90^. Furthermore, Slc12a8 requires sodium ions for NMN transport, and it is insensitive to WNK463, a specific inhibitor for the WNK kinase that regulates the activity of the cation-chloride co-transporters. Fourth, *Slc12a8* expression is upregulated in the aged ileum in response to its NAD^+^ decrease. The abrogation of *Slc12a8* upregulation has aged mice, but not young mice, fail to maintain normal NAD^+^ levels in the ileum. All these characteristics clearly demonstrate that Slc12a8 is a transporter specific to NMN.

After publishing these results, the function of Slc12a8 as a novel NMN transporter was called into question^91^. Even after we clarified the questioned points convincingly in our response^92^, one big obstacle still remained: the lack of a highly accurate, quantitative methodology for NMN measurement in biological samples. Some methodologies with liquid chromatography-tandem mass spectrometry (LC-MS/MS) were previously used to measure NMN in biological samples^31,70,93–95^. However, significant technical difficulties in sample extraction, recovery efficiency, matrix effect correction, and absolute quantification still need to be overcome. Most recently, we have finally succeeded in developing a double isotope-mediated LC-MS/MS methodology (dimeLC-MS/MS) for absolute, accurate quantification of NMN in biological samples^21^. With this new methodology, three important biological results have been obtained for the pharmacokinetic nature of NMN. First, as reported originally^15,67^, the transport of NMN into blood circulation occurs very quickly, within 5–10 min. Thus, it is completely understandable if NMN transport failed to be detected at much later time points (2–6 h)^96,97^. Indeed, one study reported no NMN detection at early time points (5–45 min) after oral and IV administrations. In this particular study, blood specimens were snap-frozen and later extracted^93^. We have already pointed out the importance of analyzing freshly prepared plasma samples to accurately detect NMN in our previous publication^15^. By extracting plasma samples freshly without freezing/thawing, we were able to detect doubly labeled, isotopic NMN (O18-D-NMN) (~20% of total NMN) in plasma at 5 min after oral gavage^15^. It has also been suggested that, via its extracellular dephosphorylation by CD73, NMN could be converted to NR, which is transported and utilized to synthesize NAD^+^ via the NR kinase (NRK) pathway^9,11,98^. Now, it has been shown clearly that there is no significant conversion from NMN to NR within the time frame when rapid NMN transport occurs^21^. Additionally, it has been reported that whereas NR is degraded quickly to nicotinamide in plasma, NMN is quite stable in this condition^98^. These results could also provide an additional explanation as to why plasma NR levels are very low at a basal level and even after NMN administration. Second, while the capability of NMN uptake in the gut decreases with age, the degradation of NMN to nicotinamide happens to a similar extent in both young and old mice^21^. This finding raises a caution for the use of very high doses of NMN in old individuals because high-dose nicotinamide has been suggested to have toxic potential and thus, unsupervised use should be discouraged^99^. Third, a significant amount of NMN can be transported directly into cells in a Slc12a8-dependent manner^21^. In the case of a mouse hepatocyte cell line AML12, NMN was transported into cells up to ~30% of the endogenous NMN pool over an hour, and this transport was dependent on Slc12a8^21^, providing definitive support to our original conclusion that Slc12a8 is an NMN transporter^15^.

The discovery of the Slc12a8 NMN transporter has brought two critical ideas to the concept of the NAD World. The first important idea is that the small intestine is the fourth key tissue that functions as a “*signal generator*” in the NAD World. Gut-specific *Slc12a8*-knockdown mice and whole-body *Slc12a8*-deficient mice show a significant reduction in NMN uptake, clearly suggesting that the small intestine, particularly the jejunum and ileum, is the major place where NMN is absorbed^15^. Interestingly, NMN exists in the luminal content of the jejunum and ileum in mice. Given that NMN is contained in vegetables, fruits, and milk^11^, the NMN detected in the small intestine may originate from food sources. However, considering that regular mouse chow does not contain NMN, it is also possible that certain species in the gut microbiome may produce NMN. Interestingly, it has been reported that normobiotic fecal microbiota transplantation significantly increases NMN levels in the serum of acute pancreatitis model mice and alleviates the severity of the disease by increasing pancreatic NAD^+^ levels^100^, strongly suggesting that normobiotic gut microbiota are capable of generating NMN and providing it to the host. While other metabolites may contribute to the effect of normobiotic fecal transplantation, it is very likely that NMN is taken up through Slc12a8 in the small intestine and brought into blood circulation in the order of minutes, and distributed to many tissues and organs, including the pancreas, throughout the body. Therefore, we speculate that the small intestine can control the uptake of NMN as a systemic signaling molecule, providing another layer of regulation for NAD^+^ homeostasis throughout the body. The second important idea is that NAD^+^ levels are regulated locally and systemically by several different feedback loops. Indeed, the expression of *Slc12a8* is regulated in response to cellular NAD^+^ levels^15^. It is upregulated in response to NAD^+^ decline, whereas it is downregulated in response to NAD^+^ increase. SIRT1 appears to be involved in the regulation of *Slc12a8* expression in the small intestine^101^, although mechanistic details remain unknown. A similar feedback mechanism might exist for NR uptake as well because *Slc12a8*-deficient primary mouse hepatocytes tend to show a higher uptake of NR compared to controls^15^. These observations strongly implicate that multiple feedback mechanisms exist to maintain tissue NAD^+^ homeostasis, and such feedback mechanisms seem to become more important in aged animals to counteract age-associated NAD^+^ decline. Thus, further investigation for mechanistic details of these feedback mechanisms will be necessary to understand how exactly NAD^+^ decline happens locally and systemically over age.

## Slc12a8 in the lateral hypothalamus contributes to the pathogenesis of age-associated sarcopenia and frailty

**Fig. 3:**
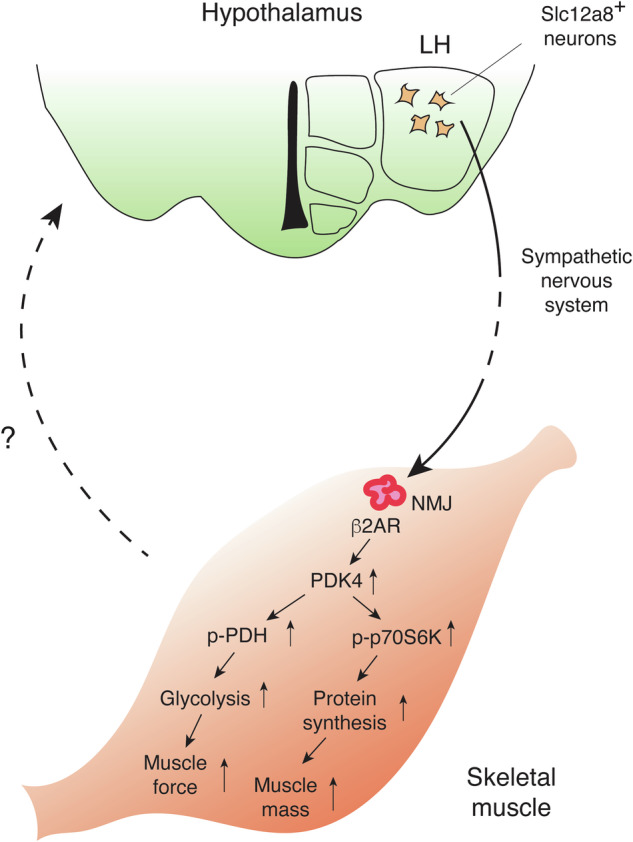
Slc12a8, a NMN transporter, in the LH is critical to maintain the structure and function of skeletal muscle during aging. Slc12a8-positive (Slc12a8^+^) neurons in the LH send a signal to neuromuscular junctions (NMJs) in skeletal muscles through the sympathetic nervous system. Sympathetic nerves innervate at NMJs and activate β2 adrenergic receptors (β2ARs), which increases the expression of pyruvate dehydrogenase kinase 4 (PDK4). Increases in PDK4 induce phosphorylation of pyruvate dehydrogenase (p-PDH) and p70S6 kinase (p-p70S6K), promoting glycolysis and protein synthesis, respectively. Muscle force and mass are maintained through this communication between the LH and skeletal muscle, counteracting age-associated sarcopenia and frailty. Skeletal muscles respond to sympathetic nervous stimulation and most likely send a reciprocal signal back to the hypothalamus. The nature of this signal is currently under intensive investigation.

Recently, another study has demonstrated the central involvement of Slc12a8 in the pathogenesis of age-associated sarcopenia and frailty^102^ (Fig. 3). The findings in this study add a new mechanism of systemic action for NMN, and thus, key findings are summarized in this section. First, Slc12a8 was found to be expressed in the lateral hypothalamus (LH) and the arcuate nucleus. Interestingly, LH-specific *Slc12a8* knockdown (KD) mice show significant decreases in energy expenditure and carbohydrate expenditure during the dark time. Because LH-specific *Nampt* KD mice were able to recapitulate very similar phenotypes to LH-specific *Slc12a8* KD mice, showing significant decreases in energy and carbohydrate expenditure, these results suggest that decreased NAD^+^ levels were most likely the cause of the phenotypes in LH-specific *Slc12a8* KD mice. Furthermore, LH-specific *Slc12a8* KD mice exhibit decreases in endurance capacity, muscle force, and fatigue resistance. Consistent with these phenotypes, LH-specific *Slc12a8* KD mice also display significant decreases in tissue weights of fast hindlimb muscles, such as the tibialis anterior (TA), gastrocnemius (GAS), and quadriceps (QUA) muscles, due to decreased phosphorylation of p70S6 kinase (p70S6K) and resultant reduction in protein synthesis. Glycolysis is also impaired in fast muscles of LH-specific *Slc12a8* KD mice, due to decreased pyruvate dehydrogenase kinase 4 (PDK4) expression and resultant reduction in phosphorylation of pyruvate dehydrogenase (PDH). These defects appear to be caused by the impairment of the sympathetic nerve-β2 adrenergic receptor (β2AR) axis in LH-specific *Slc12a8* KD mice, and similar molecular defects are also observed in skeletal muscles of aged wild-type mice. Indeed, during the process of aging, expression of *Slc12a8* in the LH decreases to an extent similar to KD efficiency in mice. Remarkably, LH-specific *Slc12a8* overexpression significantly improves energy and carbohydrate expenditure, endurance capacity, muscle mass and force, and the sympathetic nerve-β2AR axis in aged mice, ameliorating age-associated sarcopenia and frailty. These findings strongly suggest that the inter-tissue communication between the LH and skeletal muscle plays a critical role in the maintenance of skeletal muscle mass and function during the process of aging, and Slc12a8-positive LH neurons most likely mediate this particular inter-tissue communication (Fig. 3). In this case, it will be of great importance to examine whether NMN could prevent age-associated sarcopenia and frailty through the activation of Slc12a8-positive LH neurons. Given the expression of *Slc12a8* decreases in the LH over age, it will also be of great interest to develop compounds that could activate the function of Slc12a8 in those specific neurons and prevent or treat age-associated sarcopenia and frailty.

## eNAMPT-containing extracellular vesicles (eNAMPT-EVs) are another key player in mammalian aging and longevity control

While NMN is absorbed in the small intestine and distributed to various tissues through blood circulation, eNAMPT, the enzyme that produces NMN, exists in blood circulation as well in both mice and humans^19^. The function of this circulating eNAMPT has long been controversial. The NAMPT protein needs to form a dimer to function as an NAD^+^ biosynthetic enzyme^103^, and it has been reported that circulating eNAMPT forms a dimer under a normal, physiological condition^104^. On the other hand, there have been a number of papers reporting that eNAMPT functions as a proinflammatory cytokine^105^. This particular conclusion has been made mainly based on the effect of various recombinant NAMPT proteins in vitro and in vivo and has not been confirmed rigorously in loss- and gain-of-function *Nampt* mouse mutants. An important clue to resolve this long-term controversy came from the observation that monomeric eNAMPT levels increase in high-fat diet-induced type 2 diabetes model mice, whereas dimeric eNAMPT levels do not change^106^. Even in humans, individuals without type 2 diabetes have 96% and 4% of dimeric and monomeric eNAMPT, respectively, whereas individuals with type 2 diabetes show 71% and 29% of each species^106^. Administration of monomeric eNAMPT causes elevated blood glucose levels, impaired glucose tolerance, impaired glucose-stimulated insulin secretion, and certain inflammatory responses without alteration in NAD^+^ levels in vivo. Monomeric eNAMPT also impairs β cell function and induces proinflammatory genes, such as *Il1b* and *Ccl2*, in both cultured mice and human islets^106^. These findings strongly suggest that it is indeed monomeric eNAMPT that functions as a proinflammatory cytokine. For this particular function, Toll-like receptor-4 (TLR4) has been suggested to be a receptor or a binding partner for NAMPT (a.k.a PBEF)^107–109^, and a humanized anti-NAMPT monoclonal antibody has been developed to neutralize such proinflammatory cytokine function^110,111^. It should also be noted that adipose tissue-specific *Nampt* knock-in (ANKI) mice maintain 3–4-fold higher levels of circulating eNAMPT at 24 months of age, compared to those in age-matched control mice, but do not show any significant increases in circulating proinflammatory cytokines in both males and females^23^. Furthermore, supplementation with eNAMPT-EVs purified from young-to-middle-aged mice significantly extends the lifespan of aged mice, with no obvious adverse effects^23^. These genetic and pharmacological results provide strong support to distinguish the physiological function of eNAMPT-EVs from the pathological function of monomeric eNAMPT as a proinflammatory cytokine. Given that a hypothesis that monomeric eNAMPT could function as a DAMP (damage-associated molecular pattern)-like molecule was proposed back in 2009^112^, these recent findings have provided compelling evidence for this hypothesis and strong support for a pathophysiological function of monomeric eNAMPT.

**Fig. 4:**
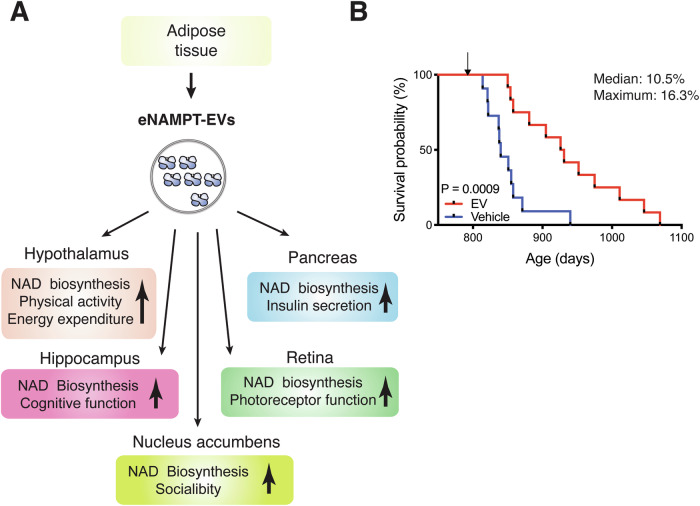
eNAMPT-containing extracellular vesicles (eNAMPT-EVs) enhance NAD^+^ biosynthesis and tissue functions in target tissues and promote lifespan in mice. **A** eNAMPT-EVs, secreted from adipose tissue, target multiple tissues, including the hypothalamus, hippocampus, nucleus accumbens, pancreas, and retina, and enhance NAD^+^ biosynthesis in these tissues and their functions, as indicated in this scheme. Currently, the mechanism by which eNAMPT-EVs target these specific tissues remains unknown. **B** Complete Kaplan–Meier curves of aged female mice administered with vehicle or EVs purified from plasma of 4- to 12-month-old mice. The lifespan results were published previously when two EV-administered mice were still alive^23^. The median and maximal lifespans were extended by 10.5% and 16.3%, respectively. Gehan-Breslow-Wilcoxon test was used for the statistical analysis of lifespan results.

Then, what is the physiological function of circulating eNAMPT? It turns out that circulating eNAMPT is almost exclusively encapsulated in extracellular vesicles (EVs) in both mice and humans^23^. The size of EVs purified from mouse and human plasma ranges from 20 to 100 nm, defining them as small EVs. These eNAMPT-containing EVs are fused to target cells, such as hypothalamic and hippocampal neurons, and eNAMPT is internalized to the cytoplasm and enhances NMN and NAD^+^ biosynthesis intracellularly^23^. On the other hand, the NAMPT protein alone cannot be internalized into the cytoplasmic fraction of cells. These findings immediately suggest that the physiological significance of EV-contained eNAMPT is to maintain NAD^+^ homeostasis systemically by enhancing NMN biosynthesis in target tissues, including the hypothalamus, hippocampus, nucleus accumbens, pancreas and retina^17,18,22,23^ (Fig. 4A). A significant fraction of circulating EV-contained eNAMPT, approximately a half of its total, is produced by adipose tissue^22^. The liver and leukocytes have been suggested to be other sources of eNAMPT^18,113,114^. In adipose tissue, intracellular NAMPT (iNAMPT) is acetylated. Among five acetylated lysines on iNAMPT, lysine 53 (K53) plays an important role in regulating the fate and the enzymatic activity of NAMPT^22^. When K53 on iNAMPT is deacetylated by adipose SIRT1, the NAMPT protein is predisposed to secretion, and its enzymatic activity is enhanced. Interestingly, another mammalian sirtuin, SIRT6, also deacetylates K369 on iNAMPT and suppresses its release from cancerous cells^115^. Thus, the fate of the NAMPT protein appears to be regulated by different sirtuins, most likely in a tissue- or cell-type-specific manner. The SIRT1/NAD^+^-dependent regulation of eNAMPT secretion from adipose tissue shows significant physiological relevance. In response to fasting, adipose eNAMPT secretion is enhanced in a SIRT1-dependent manner, maintaining hypothalamic NAD^+^ levels, SIRT1 activity, and physical activity during this metabolic deficit^22^ (Fig. 4A). Additionally, prepuberty stress causes reduced sociability in male mice through decreased eNAMPT secretion from adipose tissue and impaired NAD^+^/SIRT1 pathway in the nuclear accumbens (NAc)^17^ (Fig. 4A). Interestingly, normalization of circulating eNAMPT levels, as well as administration of NMN, significantly ameliorates stress-induced sociability deficits through restoration of NAD^+^ levels in the NAc^17^. It has also been demonstrated that circadian oscillation of circulating eNAMPT promotes hypothalamic NAD^+^ biosynthesis, SIRT2-FOXO1 axis, and locomotor activity during the dark time in mice^18^. Therefore, although the detailed mechanism of eNAMPT encapsulation into EVs still remains unclear, this EV-mediated eNAMPT delivery system plays a critical role in maintaining NAD^+^ homeostasis in multiple tissues and organs under various pathophysiological conditions.

The EV-mediated eNAMPT delivery system becomes less efficient over age, decreasing the amounts of circulating eNAMPT linearly in both mice and humans^23^. It is because NAMPT-mediated NAD^+^ biosynthesis in adipose tissue decreases over age^24^ and also because the sympathetic nervous tone, a signal necessary to have adipose tissue secrete eNAMPT-EVs, decreases over age^20^. Interestingly, the remaining lifespans of individual mice are highly correlated with their plasma eNAMPT levels, making eNAMPT a valuable surrogate biomarker of aging, at least in mice^23^. Furthermore, adipose tissue-specific *Nampt* knock-in (ANKI) mice, which maintain 3–4-fold higher plasma eNAMPT levels compared to age-matched controls, exhibit a broad range of anti-aging phenotypes, including significant enhancement in wheel-running and locomotor activities and sleep quality and improvement in glucose-stimulated insulin secretion, photoreceptor function, and cognitive function, when they become 18–20 months of age^23^ (Fig. 4A). Consistent with these phenotypes, aged ANKI mice maintain higher tissue NAD^+^ levels than age-matched controls in the hypothalamus, hippocampus, pancreas, and retina, although there are some sex-dependent differences. The hypothalamic expression of *orexin type-2 receptor* (*Ox2r*) and *PR domain 13* (*Prdm13*), two confirmed SIRT1 target genes in the hypothalamus, is upregulated in aged ANKI mice, particularly in females. Interestingly, ANKI female mice display significant extension (13.4%) of median lifespan and significant delays in age-associated mortality rate. These findings strongly suggest that the age-associated decline in circulating eNAMPT levels causally contributes to the reduction in tissue NAD^+^ levels and SIRT1 activity, leading to the physiological decline in various tissue functions and producing aging phenotypes. This age-associated functional decline can be counteracted by increasing circulating eNAMPT.

This notion has been further confirmed by transferring eNAMPT-EVs from young individuals to aged individuals. When injecting eNAMPT-EVs purified from 4–6 month-old mice into 20-month-old mice, their wheel-running activity during the dark time is significantly enhanced, whereas the activity during the light time decreases, most likely due to their better sleep^23^. This effect can be recapitulated by supplementing eNAMPT-EVs purified from the media of cultured wild-type adipocytes, but not *Nampt*-knockdown adipocytes. Most remarkably, supplementing eNAMPT-EVs purified from young-to-middle-age mice once a week for three months, starting at 26 months of age, significantly extends both median and maximal lifespans by 10.5% and 16.3%, respectively^23^ (Fig. 4B). eNAMPT-EV-administered aged mice look much healthier with shinier and richer fur and much more active, compared to vehicle-administered age-matched control mice. Most recently, another study has reproduced the anti-aging and lifespan-extending effects of EVs purified from young mice^116^. In this study, the effects of young EVs are ascribed to several microRNAs, although their in vivo significance has not been evaluated, suggesting that different components in young EVs may cooperate together to counteract aging. Taken together, these findings clearly demonstrate the importance of the EV-mediated eNAMPT delivery system in the regulation of aging and longevity in mammals and the potential of eNAMPT-EVs as an effective agent to counteract age-associated functional decline. It will also be critical to understand how eNAMPT-EV secretion is regulated in adipose tissue. If we develop compounds that can stimulate the mechanism of eNAMPT-EV secretion, such compounds could be used as another effective anti-aging intervention.

## NAD World 3.0: the importance of inter-tissue communications between four key tissues through NMN/Slc12a8 and eNAMPT-EVs

**Fig. 5:**
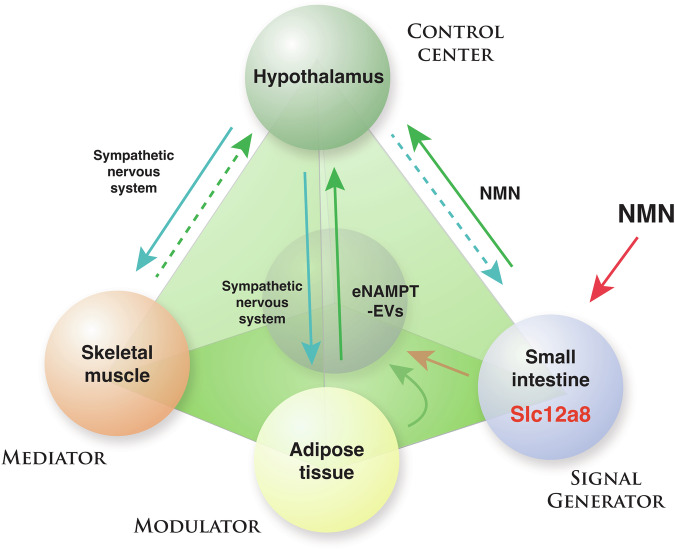
NAD World 3.0: The inter-tissue communications between four key tissues through NMN/Slc12a8 and eNAMPT-EVs control aging and longevity in mammals. In the NAD World 3.0 concept, four key tissues are proposed to play critical roles in the regulation of mammalian aging and longevity: the hypothalamus as the control center of aging, skeletal muscle as a mediator, adipose tissue as a modulator, and the small intestine as a signal generator. Between the hypothalamus and each of the three peripheral tissues, there are specific feedback loops that may interact with each other and contribute to the system dynamics of the NAD World. Different populations of hypothalamic neurons appear to regulate each feedback loop, and NMN/Slc12a8 and eNAMPT-EVs are key regulatory components in these feedback loops. Details of this new concept and important predictions are described in the text.

All these new developments described above have led to the next reformulation of the NAD World concept, resulting in the NAD World 3.0. As a key feature of the NAD World 3.0, multi-layered inter-tissue communications mediated by NMN and eNAMPT are proposed to function as core machinery for mammalian aging and longevity control (Fig. 5). Now the small intestine is added to this reformulated concept as the fourth critical tissue that functions as a “*signal generator*”. In the NAD World 3.0, the small intestine provides a gating function for the uptake of NMN and the maintenance of NMN in blood circulation (Fig. 5). In the original version of the NAD World concept, the extracellular biosynthesis of NMN by eNAMPT was hypothesized^2^. Clearly, this is not the case, given that eNAMPT is encapsulated into EVs and that neither nicotinamide nor NMN is detected in EVs purified from plasma^23^. Therefore, it is very likely that NMN in blood circulation is not the product of the extracellular enzymatic reaction. Instead, we now hypothesize that NMN in blood circulation is maintained by the uptake of NMN through Slc12a8 in the small intestine. Recently, it has been reported that NMN is produced by lactic acid bacteria (LAB) that belong to the genus *Fructobacillus*^117^. Interestingly, these LAB seem to use NAMPT to synthesize NMN directly from nicotinamide. In honey bees, these LAB provide benefits, such as antibacterial activity, to their hosts^118^. Although the source of NMN in the mammalian small intestine still remains unknown, it will be of great interest to examine whether some bacterial species produce NMN in the small intestine, controlling NAD^+^ biosynthesis in their hosts.

In the new NAD World 3.0 concept, each inter-tissue communication is proposed to be mediated by either NMN/Slc12a8- or eNAMPT-EV-driven NAD^+^ biosynthesis (Fig. 5). Then, a critical question is: What is the difference in their physiological roles between NMN/Slc12a8-driven NAD^+^ biosynthesis and eNAMPT-EV-driven NAD^+^ biosynthesis? One potential explanation could be that NMN/Slc12a8-driven NAD^+^ biosynthesis aims to regulate cellular functions relatively quickly (an order of minutes-to-hours), whereas eNAMPT-EV-driven NAD^+^ biosynthesis provides regulation in a much longer time scale (an order of hours-to-days). The short-term vs. long-term demands of NAD^+^ biosynthesis could also be tissue-dependent. For example, the liver, which expresses a fair amount of Slc12a8, responds quickly to the increase in NMN, initiating NAD^+^ biosynthesis immediately, whereas it does not show significant NAD^+^ increases in response to administration of purified eNAMPT-EVs^16^. Contrarily, the hypothalamus, particularly DMH, shows significant increases in NAD^+^ levels in response to eNAMPT-EVs^16^. Thus, differential utilization of these two NAD^+^ biosynthesis systems might comprise distinct, time-sensitive regulations of NAD^+^ biosynthesis in key tissues and organs that are involved in mammalian aging and longevity control. To further test this hypothesis, there are two important mechanisms to understand: (1) The mechanism by which *Slc12a8* expression is regulated in response to NAD^+^. It has already been shown that the *Slc12a8* mRNA expression is induced by decreasing NAD^+^^15^, and SIRT1 might be involved in this regulation^101^. Thus, it will be important to understand the kinetics and tissue-dependency of this specific regulation. (2) The mechanism by which eNAMPT-EVs are delivered to specific tissues. Based on NAD^+^ increases observed in ANKI mice, eNAMPT-EVs appear to be targeted to specific tissues. However, because no such mechanism has been identified so far, how eNAMPT-EVs can target specific tissues is an open mystery that needs to be answered. We are currently speculating a receptor that could recognize eNAMPT-EVs specifically and mediate their uptake. Fundamentally, we still do not understand why some specific tissues rely on eNAMPT to maintain their NAD^+^ levels. Nonetheless, once we can successfully identify the mechanism of eNAMPT-EV recognition, we may be able to better understand the evolutional necessity of the eNAMPT-EV-dependent NAD^+^ regulation. Therefore, it will be critical to identify such new regulatory machinery for Slc12a8 and eNAMPT-EVs and analyze the time-dependent regulations of NMN or eNAMPT-EV uptake in each tissue.

Lastly, it should be emphasized that both NAD^+^ biosynthesis systems target the hypothalamus, the control center of aging in mammals^6^, suggesting that maintaining proper NAD^+^ levels in the hypothalamus is essential to maintain biological robustness and counteract aging. It is very likely that target neuronal populations are different between NMN/Slc12a8-driven NAD^+^ biosynthesis and eNAMPT-EV-driven NAD^+^ biosynthesis. Furthermore, each target neuronal population most likely comprises a unique feedback loop between the hypothalamus and a specific peripheral tissue, such as adipose tissue, skeletal muscle, and small intestine (Fig. 5). Most recently, we have successfully demonstrated such a feedback loop between the hypothalamus and white adipose tissue^20^. A newly identified subset of neurons in the DMH, called DMH^Ppp1r17^ neurons, regulates the function of white adipose tissue through the sympathetic nervous system, stimulating lipolysis and promoting eNAMPT secretion. Remarkably, the genetic and chemogenetic activation of DMH^Ppp1r17^ neurons significantly ameliorates age-associated dysfunction in white adipose tissue, increases circulating eNAMPT levels and physical activity, reduces age-associated mortality rate, and then extends lifespan in mice^20^, demonstrating that this hypothalamic-adipose inter-tissue communication plays a key role in mammalian aging and longevity control (Fig. 5; blue and green lines between the hypothalamus and adipose tissue). Compared to the inter-tissue communication mediated by eNAMPT-EV-driven NAD^+^ biosynthesis, further investigation will be necessary for the inter-tissue communication mediated by NMN/Slc12a8-driven NAD^+^ biosynthesis. Intriguingly, significantly high concentrations of NMN, ~10 μM on average, have most recently been detected in human breast milk by LC-MS/MS^119^. The measured concentrations of NMN are much higher than those of NAD^+^, nicotinamide, and NR, suggesting that NMN is a major NAD^+^ precursor in human breast milk. Importantly, among 150 randomly selected mother-infant pairs, there is a significant positive correlation between NMN and all developmental indicators for infants at 24 months^119^. These findings raise an interesting possibility that infants uptake NMN from their mothers’ breast milk and utilize the NMN/Slc12a8-mediated NAD^+^ biosynthesis system for their development. Another interesting place where the NMN/Slc12a8-mediated NAD^+^ biosynthesis system looks important is the inter-tissue communication between the LH, Slc12a8-positive LH neurons in particular, and skeletal muscle^102^ (Fig. 5, a blue line from the hypothalamus to skeletal muscle). Currently, a path from the skeletal muscle back to the hypothalamus is under intensive investigation (Fig. 5, a green dotted line from the skeletal muscle to the hypothalamus). The kinetic nature and the robustness or the fragility of these multi-layered feedback loops basically define the system dynamics of the NAD World 3.0, regulating the process of aging and eventually determining lifespan. In this regard, near-future investigations should identify the target neuronal populations and manipulate the activity of those specific neurons by opto- or chemogenetics to fully understand the physiological significance of such intertwined feedback loops in mammalian aging and longevity control. Once we fully elucidate how each key inter-tissue communication works, we should be able to start developing effective anti-aging interventions that target and control the main regulatory factors in each feedback loop, such as NMN and eNAMPT-EVs. The NAD World 3.0 can now provide the basic foundation for the management of these key inter-tissue communications to maintain the robustness of our physiological system against many metabolic and environmental perturbations over time.

## Concluding remarks

The NAD World 3.0 is an attempt to understand a comprehensive image of a big, complicated elephant, that is, the systemic regulatory mechanism of mammalian aging and longevity. We could touch only one place at a time. Nonetheless, taking NAD+ as a common thread, we are now able to stitch several important pieces of knowledge together and begin to understand the multi-layered feedback loops between the hypothalamus and several key peripheral tissues, including adipose tissue, skeletal muscle, and the small intestine. By exploring the NAD World 3.0 deeper, we could also develop effective anti-aging interventions, using NMN, Slc12a8, and eNAMPT-EVs and targeting each feedback loop. Indeed, we have successfully conducted the first human clinical trial on NMN and demonstrated its efficacy in improving skeletal muscle insulin sensitivity and remodeling capability^25^, although this effect needs to be reproduced independently. Additionally, it has also been demonstrated that NAD^+^ is one of the key metabolite signatures of lifespan-extending interventions^120^. The NAD World 3.0 will provide new directions of research to understand the systemic regulatory mechanism for aging and longevity in mammals and shed new light on this big but fascinating elephant.

## Data Availability

All data supporting the findings described in this review article are available within each paper cited, and its supplementary materials and some of them are accessible through hyperlinks and persistent identifiers of the papers. Additional information could be requested from the corresponding author.
